# Supplementary material for: The causal relationship between the human gut microbiota and pyogenic arthritis: a Mendelian randomization study
Source: Front Cell Infect Microbiol. 2024 Nov 26;14:1452480. doi: 10.3389/fcimb.2024.1452480 (PMC11629706; doi:10.3389/fcimb.2024.1452480)
Supplement: Supplementary file 1 [file DataSheet1.zip › mendelian test/ebi-a-GCST90027638.csv_forest.pdf]

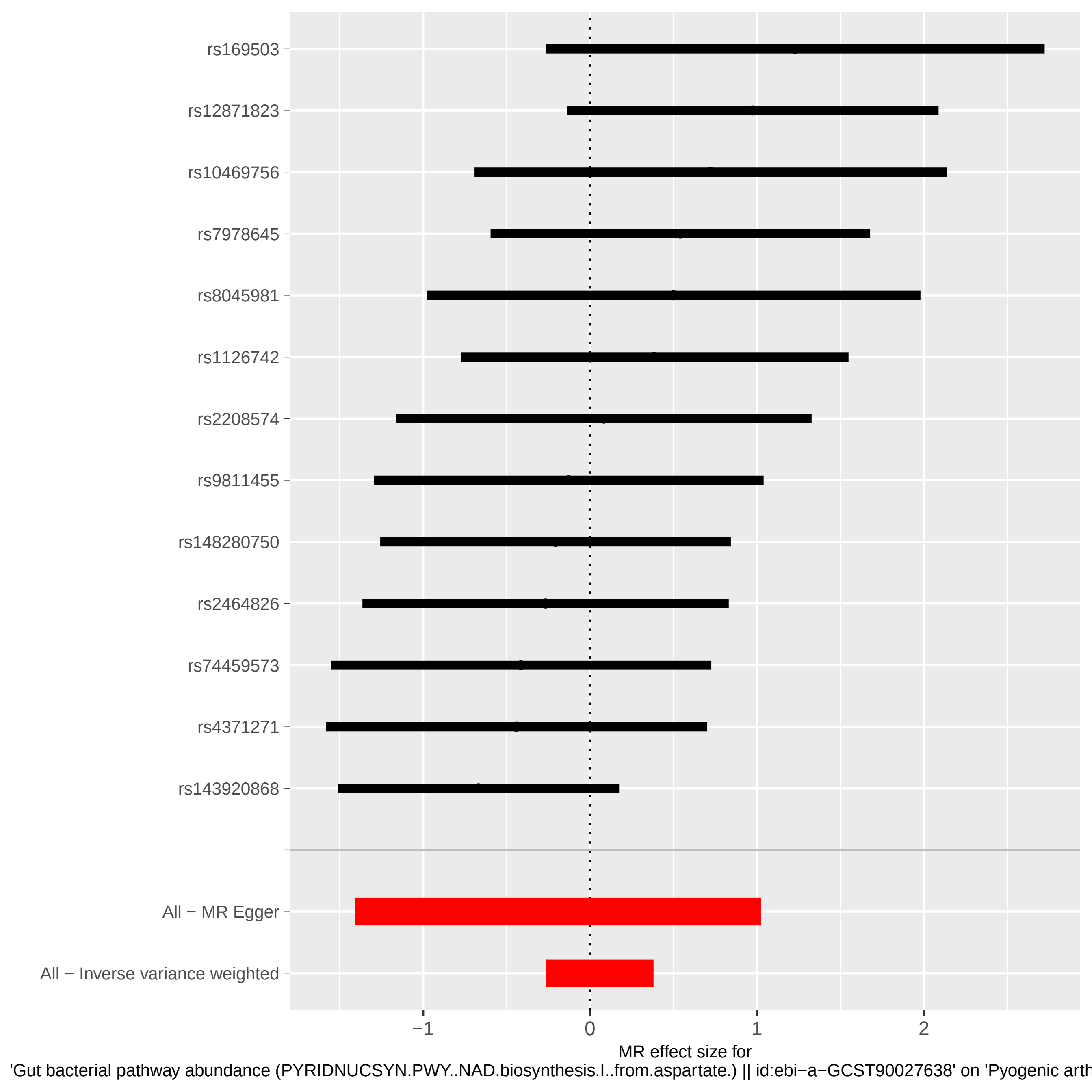
rs169503
rs12871823
rs10469756
rs7978645
rs8045981
rs1126742
rs2208574
rs9811455
rs148280750
rs2464826
rs74459573
rs4371271
rs143920868
All − MR Egger
All − Inverse variance weighted
−1
0
1
2
MR effect size for
'Gut bacterial pathway abundance (PYRIDNUCSYN.PWY..NAD.biosynthesis.I..from.aspartate.) || id:ebi−a−GCST90027638' on 'Pyogenic arth
